# Elevated first-trimester neutrophil elastase and proteinase 3 increase the risk of gestational diabetes mellitus and adverse fetal outcomes

**DOI:** 10.1186/s12958-023-01170-x

**Published:** 2024-01-02

**Authors:** Lihong Wang, Zhoujunhao Zhou, Xinming Xu, Yue Li, Rui Zhang, Zhiyan Yu, Xinmei Huang, Shufei Zang, Tiange Sun

**Affiliations:** Department of Endocrinology, Shanghai Fifth People’s Hospital, Fudan University, 801 Heqin Road, 200240 Shanghai, China

**Keywords:** Gestational diabetes mellitus, Neutrophil elastase, Proteinase 3, Macrosomia, Inflammation

## Abstract

**Background:**

Chronic inflammation plays a vital role in the development of gestational diabetes mellitus (GDM). Studies in mouse models show that neutrophil serine proteases (NSPs), neutrophil elastase (NE) and proteinase-3 (PR3) are important drivers of chronic inflammation with consequent metabolic disturbances. This study evaluated the association of NE and PR3 with GDM development and adverse fetal outcomes.

**Method(s):**

This was a prospective cohort study. Serum PR3 and NE concentration was measured in all enrolled pregnant women in the first and the second trimester to determine the connection between NSPs and GDM and adverse fetal outcomes. Logistic regression, spline regression and linear regression analyses were applied to investigate the association of NE or PR3 with GDM development and adverse fetal outcomes. The concentration of NE and PR3 in placental biopsies was evaluated by semi-quantitative analysis of immunohistochemistry staining.

**Result(s):**

NE or PR3 concentration in the first trimester, rather than the second, increased more significantly in women with GDM than in those without, regardless of pre-pregnancy body mass index and age. There was a stepwise increase in GDM occurrence as well as comprehensive adverse fetal outcomes across tertiles of NE and PR3. NE and PR3 were positively associated with neutrophil count, pre-pregnancy BMI, plasma glucose level and newborn weight. Logistic regression revealed NE or PR3 to be independent risk factors for the development of GDM and comprehensive adverse fetal outcomes. Spline regression showed a significant increased risk of GDM occurrence and comprehensive adverse fetal outcomes when serum NE concentration exceeded 417.60 ng/mL and a similar result for PR3 and GDM occurrence when the latter exceeded 88.52 ng/mL. Immunohistochemistry data confirmed that enriched NE and PR3 content in placental tissue may have contributed to the development of GDM.

**Conclusion(s):**

This work demonstrates that excessive first-trimester NE and PR3 increase the risk of GDM development and comprehensive adverse fetal outcomes.

**Supplementary Information:**

The online version contains supplementary material available at 10.1186/s12958-023-01170-x.

## Introduction

 Gestational diabetes mellitus (GDM) has been defined as an abnormal glucose increase at onset or first recognition during pregnancy [1]. The incidence of GDM has risen with economic development and improved living standards as well as increased screening for GDM [1]. Gestational diabetes is a common chronic condition during pregnancy that damages the health of millions of women worldwide [2, 3]. According to a meta-analysis, the overall incidence of GDM in the Chinese mainland is 14.8% [4]. Over the last few years, the prevalence of GDM has been strongly associated with obstetric and neonatal complications including preeclampsia, need for cesarean section, and macrosomia (neonatal birthweight > 4000 g) [1]. Although multiple factors (including family or personal history of diabetes mellitus, previous adverse pregnancy outcomes, diabetes mellitus, and obesity) have been associated with GDM development [5], its exact pathophysiology remains unclear.

Many studies have shown that inflammation plays a pivotal role in the progression of GDM. Once pregnant, the body gradually enters a state of low-grade systemic inflammation [6–9]. Abnormal elevation of white blood cell (WBC) count, neutrophil count and neutrophil-to-lymphocyte ratio are simple markers of inflammation [5]. Their ability to predict GDM was investigated in our previous retrospective study that demonstrated first-trimester neutrophil count to be closely associated with the development of GDM and adverse pregnancy outcomes [5]. Nonetheless how neutrophils mediate development of GDM and through which effector substances is not clear. Neutrophil elastase (NE) and proteinase-3 (PR3) are the two main neutrophil serine proteases (NSPs) that are stored in their active form in nitrogenophil granules and are not released until the neutrophils are specifically stimulated [10]. NE plays an important role in insulin resistance and metabolic inflammation. When NE and PR3 are activated, in addition to their microbiocidal activity, they play an important role in non-infectious inflammation such as diabetic atherosclerosis [11]. One study reported that obese mice and human subjects had increased activity of NE. NE null (Ela2−/−) mice showed a significantly reduced inflammatory response and increased insulin sensitivity [12]. We speculated that NE may act as an important effector of neutrophils in the occurrence and development of GDM. This study aimed to investigate the potential correlation of NSPs with GDM and adverse fetal outcomes.

## Research design and methods

All subjects were registered from May 2018 to May 2023 at the GDM Care Center of Shanghai Fifth People’s Hospital, Fudan University. During the first antenatal visit at around 8–14 weeks of gestation, a baseline questionnaire was administered to record information about obstetric history, family history of diabetes, previous history of GDM, method of conception, parity, and pre-pregnancy weight. Gestational age was determined based on the date of the last menstrual period or by an early pregnancy ultrasound examination. Pre-pregnancy BMI was calculated as pre-pregnancy weight in kilograms divided by the square of height in meters. Blood was collected after fasting for 12 h overnight for routine (Automatic Blood cell analyzer, Sysmex XN9000, Japan) and biochemical analysis (Automatic biochemical analyzer, Roche Cobas 8000, Switzerland) and blood pressure measured at each visit. In addition, participants signed an informed consent form. Follow-up surveys were conducted when an OGTT was performed and at delivery. After delivery, placental tissue was collected.

**Fig. 1:**
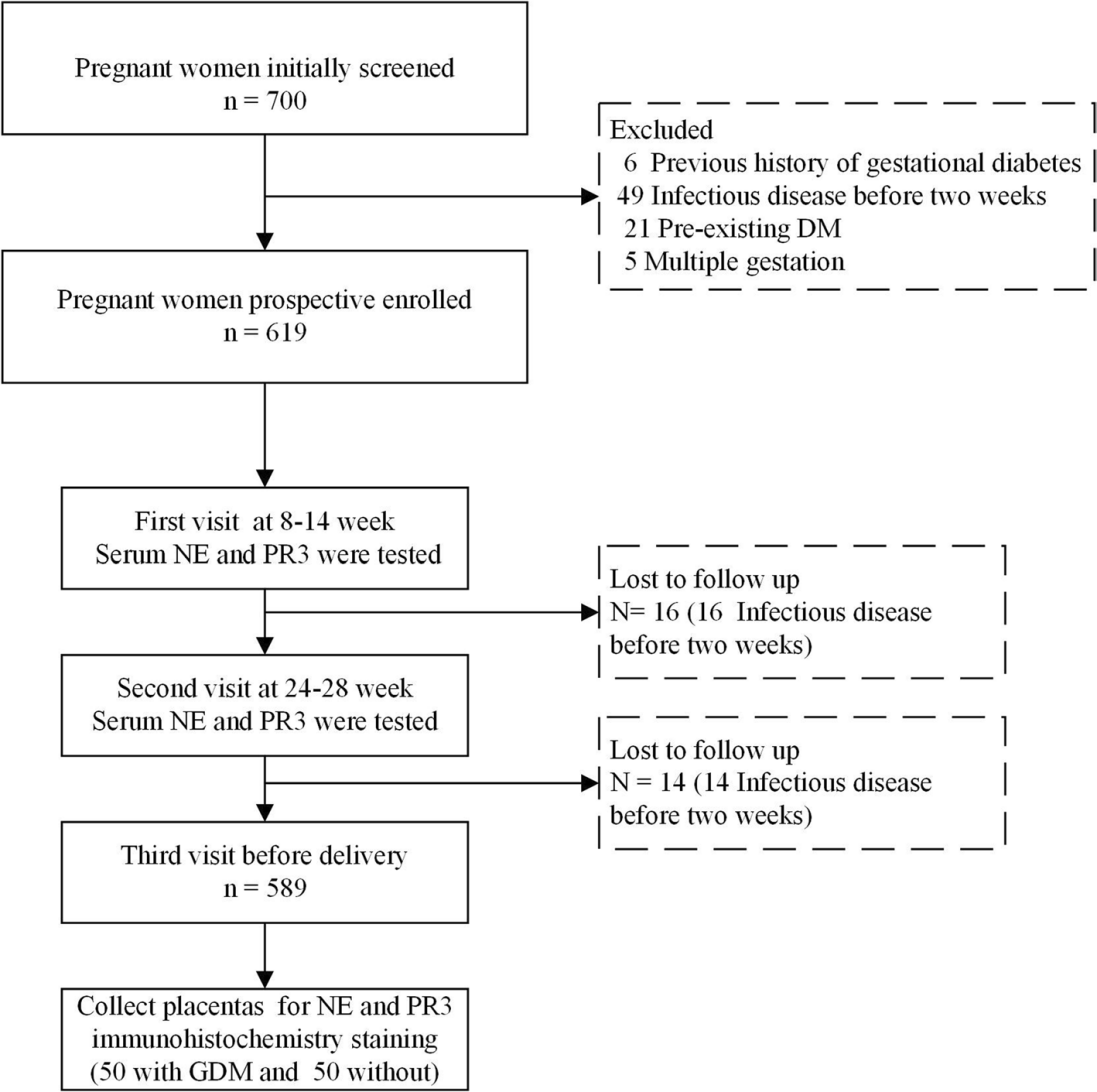
A flow chart of the steps to obtain the final study sample

Women were excluded if they had any of the following: (1) previous history of gestational diabetes; (2) infectious disease in the two weeks preceding baseline blood tests; (3) abnormal liver or renal function; (4) presence of viral infection or positive carrier status (hepatitis B virus, syphilis or HIV); (5) pre-existing DM; (6) chronic hypertension; (7) multiple gestation. A flow chart of the steps to obtain the final study sample is shown in Fig. 1.

### Placental tissue specimen

Placental tissue was collected from 50 women with GDM and 50 women without, matched for pre-pregnancy BMI and age. All tissue was formalin-fixed and stained with hematoxylin-eosin (H&E).

### Semi-quantitative analysis of immunohistochemistry staining

Serum NE and PR3 level in placental tissue biopsy sections was determined by a semi-quantitative analysis of immunohistochemistry staining. Formalin-fixed paraffin-embedded placental tissue with GDM or without was selected for immunohistochemical staining with antibody against human NE (Abcam, no. 68672, 1:50) and human PR3 (SCB, no. K3020, 1:100). Staining was visualized by ImageJ under 20X magnification and the average NE- or PR3-positive cells in each section calculated to compare average density (average optical density, AOD) of staining.

### Exposures

Serum NE and PR3 were tested (BioVendor, Germany) at baseline and during the OGTT.

### Outcomes

The outcomes of interest in this study were GDM and comprehensive adverse fetal outcomes. All enrolled pregnant women completed routine screening for GDM at 24 to 28 weeks’ gestation by a 75 g OGTT [13]. OGTT was performed in the morning after an overnight fast of at least 8 h and GDM diagnosed when fasting plasma glucose (FPG) was ≥ 5.1 mmol/L, 1 h plasma glucose (1 h PG) ≥ 10.0 mmol/L or 2 h plasma glucose (2 h PG) ≥ 8.5 mmol/L. An OGTT area was crudely calculated as 1/2 (FPG + 1/2 1 h PG) + 1/2 (1/2 1 h PG + 2 h PG).

After delivery, detailed information such as gestational age, delivery method, newborn weight, and gender was recorded. Macrosomia was defined as newborn weight above 4000 g. Comprehensive adverse fetal outcomes were identified as macrosomia, low Apgar score (< 7) and prematurity.

### Statistical analysis

Analysis of variance (ANOVA) and Student’s t-test were used to identify difference in mean between groups. Bonferroni correction was applied for multiple comparisons. Non-normally distributed variables were analyzed by Kruskal-Wallis or Wilcoxon tests. NE and PR3 were Log transformed for t testing. A case-control matching method was employed to match variables that included pre-pregnancy BMI and age. Matching tolerance was 2 and 2 respectively. Further comparison was made of NE and PR3 between women with GDM and women without.

In this cohort study, to strengthen an association of NE or PR3 with the occurrence of GDM and adverse fetal outcomes, patients were divided into three groups based on their tertile of NE and PR3: NE lowest group (< 311.49 ng/mL), middle (311.49 to 514.96 ng/mL) and highest (> 514.96 ng/mL), PR3 lowest group (< 70.50 ng/mL), middle (70.50 to 111.02 ng/mL) and highest (> 111.02 ng/mL).

Descriptive statistics for studied variables are presented as mean ± standard deviation (SD) for normally distributed variables, median (interquartile range (IQR)) for non-normally distributed variables, and frequency (percentage) for categorical variables. Linear correlation between NE or PR3 and pre-pregnancy BMI, 1 h plasma glucose (1 h PG), 2 h plasma glucose (2 h PG), OGTT area, neutrophil count and newborn weight was assessed by correlation analysis. Continuous association of NE and PR3 with GDM occurrence was determined by spline regression analysis. To determine whether NE and PR3 were independent risk factors, logistic regression analysis was performed with GDM (presence/absence) or comprehensive adverse outcomes (presence/absence) classified in a binary manner as a dependent variable. All data were analyzed using SPSS 25.0 (IBM SPSS lnc, Chicago, IL, USA). Two-tailed *P* < 0.05 was considered to indicate statistical significance.

With occurrence of GDM as the outcome, it was presumed that the incidence rate of GDM with higher NE concentration would be 0.4, the incidence rate with lower NE concentration would be 0.2, with α 0.025, and 1-β 0.8. The sample size of the test group and control group was calculated using PASS 11 software, and a total required sample 194 patients obtained. Our study sample exceeded 194.

## Results

### Characteristics of all women with or without GDM and matched case-control study

A total of 589 pregnant women completed follow-up from early pregnancy to delivery of whom 96 developed GDM (incidence rate 16.3%). The cesarean section rate of pregnant women with comprehensive adverse fetal outcomes was significantly higher than that of pregnant women without (51.1% vs. 33.5%, *P* = 0.016) (Supplemental Table 1). The higher risks for preeclampsia were excluded, for example pre-existing DM and chronic hypertension. The incidence of preeclampsia and gestational hypertension was not high. Only one patient with GDM and two without who suffered preeclampsia and five with GDM and 15 without who were affected by gestational hypertension were detected (*P* > 0.05). In the first trimester, compared with women without GDM, those with GDM had a much higher weight, WBC, neutrophil count, triglyceride level and FPG (all *P* < 0.05), but much shorter height and blood urea nitrogen and high-density lipoprotein level (all *P* < 0.05). More importantly, both NE and PR3 concentration were increased significantly in women with GDM compared with those without (*P* < 0.001; *P* = 0.014 respectively). In the second trimester, women with GDM had higher, FPG, 1 h PG, 2 h PG and OGTT area (all *P* < 0.05). Although NE and PR3 in the second trimester tended to increase in those with GDM, there were no significant differences between the two groups. Patients with GDM had much higher FPG in the third trimester and lower total weight gain. In addition, a higher occurrence of comprehensive adverse fetal outcomes (10.1% vs. 23.0%, *P* = 0.001) was evident in women with GDM (Table 1).

**Table 1:** Characteristics of women with GDM and without in all subjects and matched subjects

	All subjects		*P*	Matched case - control subjects		*P*
	Women without GDM	Women with GDM		Women without GDM	Women with GDM	
Anthropometric parameters						
N	493	96	/	91	91	/
Age (years)	30.29 ± 4.41	31.24 ± 4.60	**0.040**	30.91 ± 4.42	31.04 ± 4.41	0.798
Parity (n, %)			0.368			0.628
Nulliparous	142 (35.4)	29 (30.5)		26 (28.6)	29 (31.9)	
Parous	259 (64.6)	66 (69.5)		65 (71.4)	62 (68.1)	
Pre-pregnancy BMI (kg/m^2^)	21.69 ± 3.08	23.44 ± 3.99	**< 0.001**	22.87 ± 2.96	22.94 ± 3.00	0.658
**First trimester**						
Gestation weeks	10.93 ± 3.11	11.29 ± 3.68	0.63	11.63 ± 4.21	11.23 ± 3.68	0.211
SBP (mmHg)	116.37 ± 10.86	117.61 ± 10.05	0.220	118.19 ± 9.73	117.25 ± 10.04	0.401
DBP (mmHg)	71.02 ± 8.53	71.44 ± 9.17	**0.011**	72.09 ± 8.59	71.16 ± 9.26	0.467
Weight (kg)	57.44 ± 8.58	60.68 ± 9.60	**< 0.001**	60.51 ± 8.05	59.94 ± 8.74	0.785
Height (cm)	1.60 ± 0.05	1.59 ± 0.06	**0.030**	1.60 ± 0.04	1.59 ± 0.05	0.318
WBC (× 10^9^/L)	8.62 ± 2.05	8.98 ± 1.78	**0.030**	8.61 ± 2.22	8.95 ± 1.82	**0.044**
Neutrophils (× 10^9^/L)	6.27 ± 1.40	6.81 ± 1.46	**0.005**	6.21 ± 1.42	6.80 ± 1.49	**0.008**
ALT (U/L)	10.00 (8.00–15.00)	10.00 (8.00–14.75)	0.950	11.00 (8.00–15.75)	10.00 (8.00–14.00)	0.452
BUN (mmol/L)	2.60 (2.10–3.10)	2.44 ± 0.59	**0.010**	2.61 ± 0.74	2.43 ± 0.61	0.182
SCR (umol/L)	51.57 ± 9.29	50.02 ± 7.79	0.190	51.61 ± 8.72	50.19 ± 7.87	0.267
TC (mmol/L)	4.39 (3.95–4.98)	4.49 (4.01–4.90)	0.750	4.56 ± 0.75	4.46 ± 0.69	0.385
TG (mmol/L)	1.34 (1.07–1.75)	1.62 (1.25–2.00)	**< 0.001**	1.55 (1.19–2.06)	1.59 (1.24–2.02)	0.792
HDL (mmol/L)	1.82 (1.59–2.04)	1.79 (1.51–1.99)	**0.040**	1.78 ± 0.35	1.74 ± 0.36	0.683
LDL (mmol/L)	2.48 ± 0.69	2.47 ± 0.59	0.610	2.56 ± 0.65	2.46 ± 0.59	0.455
FPG (mmol/L)	4.40 (4.14–4.62)	4.47 (4.22–4.81)	**0.020**	4.35 (4.16–4.56)	4.47 (4.22–4.79)	**0.021**
eGFR (CDK-EPI) (mL/min/1.73m^2^)	124.24 ± 10.63	124.39 ± 8.27	0.780	123.77 ± 9.97	124.42 ± 8.41	0.595
eGFR (MDRD) (mL/min/1.73m^2^)	134.50 ± 29.90	136.82 ± 24.77	0.390	133.81 ± 27.90	136.54 ± 25.02	0.306
NE (ng/mL)^a^	2.58 ± 0.26	2.70 ± 0.24	**< 0.001**	2.57 ± 0.24	2.69 ± 0.23	**< 0.001**
PR3 (ng/mL)^a^	1.94 ± 0.23	2.00 ± 0.21	**0.014**	1.93 ± 0.21	2.00 ± 0.21	**0.040**
**Second trimester**						
Gestation weeks	25.90 ± 1.40	25.81 ± 1.74	0.75	25.79 ± 1.52	25.80 ± 1.77	0.467
NE (ng/mL)	476.06 (349.26–839.20)	602.29 (429.80–760.82)	0.391	437.19 (349.05–974.50)	542.65 (359.41–704.15)	0.706
PR3 (ng/mL)	91.50 (67.83–117.19)	95.23 (68.06–138.40)	0.280	99.14 (69.94–174.84)	92.18 (63.47–135.90)	0.606
FPG (mmol/L)	4.35 ± 0.37	4.84 ± 0.58	**< 0.001**	4.39 (4.15–4.51)	4.66 (4.38–5.26)	**< 0.001**
1 h PG (mmol/L)	7.01 ± 1.59	9.86 ± 1.59	**< 0.001**	7.18 ± 1.47	9.90 ± 1.60	**< 0.001**
2 h PG (mmol/L)	6.01 ± 1.12	8.19 ± 1.59	**< 0.001**	6.07 ± 1.00	8.18 ± 1.61	**< 0.001**
OGTT area	12.18 ± 2.04	16.38 ± 2.18	**< 0.001**	12.40 ± 1.80	16.41 ± 2.20	**< 0.001**
WBC (× 10^9^/L)	9.52 ± 2.27	9.66 ± 1.95	0.250	9.49 ± 2.21	9.60 ± 1.93	0.441
Neutrophils (× 10^9^/L)	7.03 ± 1.98	7.12 ± 1.62	0.270	6.98 ± 1.96	7.08 ± 1.61	0.365
**Third trimester**						
Delivery time (weeks)	39.0 ± 1.46	38.5 ± 1.54	0.003	38.8 ± 1.58	38.5 ± 1.56	0.078
HBA1C (%)	5.41 ± 0.43	5.62 ± 0.46	0.180	5.63 ± 0.26	5.62 ± 0.48	0.830
FPG (mmol/L)	4.02 ± 0.65	4.43 ± 0.69	**< 0.001**	3.83 ± 0.43	4.40 ± 0.70	**0.002**
Weight gain (kg)	12.73 ± 4.52	9.99 ± 4.98	**< 0.001**	11.98 ± 4.52	10.00 (7.00–13.50)	**0.013**
**Pregnancy outcomes**						
Newborn weight (kg)	3.27 ± 0.45	3.32 ± 0.53	0.090	3.24 ± 3.91	3.33 ± 4.77	**0.042**
Macrosomia (n, %)			0.403			0.660
No	302 (95.6)	69 (93.2)		68 (97.1)	68 (95.8)	
Yes	14 (4.4)	5 (6.8)		2 (2.9)	3 (4.2)	
Fetus gender (n, %)			0.282			0.935
Male	143 (45.3)	39 (52.7)		36 (51.4)	37 (52.1)	
Female	173 (54.7)	35 (47.3)		34 (48.6)	34 (47.9)	
Comprehensive adverse fetal outcomes (n, %)			**0.001**			0.058
No	284 (89.9)	57 (77.0)		64 (91.4)	57 (80.3)	
Yes	32 (10.1)	17 (23.0)		6 (8.6)	14 (19.7)	
Cesarean section (n, %)			0.082			0.592
No	212 (67.1)	42 (56.8)		42 (60)	40 (56.3)	
Yes	104 (32.9)	32 (43.2)		28 (40)	31 (43.7)	
Preeclampsia (n, %)			0.532			0.316
No	491 (99.6)	95 (99.0)		91 (100)	90 (98.9)	
Yes	2 (0.4)	1 (1.0)		0 (0)	1 (1.1)	
Gestational hypertension (n, %)			0.498			0.470
No	386 (96.3)	91 (94.8)		88 (96.7)	86 (94.5)	
Yes	15 (3.7)	5 (5.2)		3 (3.3)	5 (5.5)	

Data are shown as mean ± SD, median (interquartile range) or *n* (%)

*WBC* white blood cell

^a^Log transformed for t test

A 1:1 case-control matching procedure was performed to avoid the potential bias of covariates that were not evenly distributed between those with GDM and those without, yielding 91 women with GDM and 91 without. After matching for age and pre-pregnancy BMI, there remained a significantly higher NE (*P* < 0.001) and PR3 (*P* = 0.040) concentration as well as WBC, neutrophil count and FPG in the first trimester, FPG, 1 h PG, 2 h PG in the second trimester, OGTT area, FPG in the third trimester and newborn weight (all *P* < 0.05) in those with GDM (Table 1).

### The occurrence of GDM and comprehensive adverse fetal outcomes increased across three groups categorized by tertile of NE and PR3

Subjects were divided into three groups according to tertile of NE and PR3 in the first trimester: NE (Table 2) and PR3 (Table 3) were categorized as lowest, middle or highest. There was a stepwise increase in WBC (*p* < 0.001) and neutrophil count (*p* < 0.001) in the first trimester across tertitles of NE **(**Table 2**)**. The same applied to WBC count (*p* < 0.001) and neutrophil count (*p* = 0.001) in PR3 (Table 3). More importantly, the occurrence of GDM (9.1% vs. 17.0% vs. 24.3%, *p* < 0.001) and comprehensive adverse outcomes (3.95% vs. 10.4% vs. 9.8%, *p* = 0.016) increased across tertiles of NE (Table 2) and the occurrence of GDM (12.4% vs. 14.2% vs. 23.0%, *p* = 0.011) across tertiles of PR3 (Table 3). In addition, 1 h PG gradually increased with tertile of NE (Table 2), and 1 h PG, 2 h PG and OGTT area with tertile of PR3 (all *p* < 0.05) (Table 3).

**Table 2:** Comparison of parameters among three groups categorized by tertile of NE in the cohort study

	Lowest	Middle	Highest	*P*
NE (ng/mL)	Below 311.49	311.49 to 514.96	Above 514.96	
N	196	196	197	
**Anthropometric parameters**				
Age (years)	30.81 ± 4.53	30.55 ± 4.44	30.04 ± 4.38	0.052
Parity (n, %)				0.445
Nulliparous	65 (42.2)	43 (31.4)	66 (35.3)	
Parous	89 (57.8)	94 (68.6)	121 (64.7)	
Pre-pregnancy BMI (kg/m^2^)	21.79 ± 3.10	22.04 ± 3.33	22.21 ± 3.54	0.810
**First trimester**				
Gestation weeks	11.00 ± 3.43	10.96 ± 3.13	11.04 ± 3.16	0.222
SBP (mmHg)	116.68 ± 10.58	115.51 ± 10.05	117.45 ± 11.30	0.220
DBP (mmHg)	70.84 ± 8.10	69.56 ± 8.78	72.56 ± 8.79	**0.011**
Weight (kg)	58.21 ± 8.79	57.62 ± 8.72	58.30 ± 9.07	0.682
Height (cm)	1.61 ± 0.04	1.60 ± 0.05	1.60 ± 0.06	**0.015**
WBC (× 10^9^/L)	8.05 ± 1.72	8.80 ± 1.85	9.12 ± 2.21	**< 0.001**
Neutrophils (× 10^9^/L)	5.90 ± 1.33	6.46 ± 1.36	6.69 ± 1.44	**< 0.001**
ALT (U/L)	10.00 (8.00–15.00)	10.00 (8.00–15.00)	11.00 (8.00–15.00)	0.983
BUN (mmol/L)	2.40 (2.10–3.00)	2.63 ± 0.72	2.66 ± 0.67	0.160
SCR (umol/L)	50.21 ± 8.52	51.22 ± 9.79	52.17 ± 8.68	0.083
TC (mmol/L)	4.44 ± 0.83	4.48 ± 0.75	4.57 ± 0.85	0.424
TG (mmol/L)	1.53 ± 0.75	1.50 ± 0.58	1.57 ± 0.63	0.411
HDL (mmol/L)	1.82 ± 0.36	1.84 ± 0.34	1.83 ± 0.37	0.664
LDL (mmol/L)	2.39 ± 0.71	2.46 ± 0.62	2.56 ± 0.68	0.141
FPG (mmol/L)	4.45 ± 0.38	4.36 ± 0.34	4.40 ± 0.51	0.095
eGFR (CDK-EPI) (mL/min/1.73m^2^)	125.13 ± 9.59	124.32 ± 11.48	123.52 ± 9.49	0.305
eGFR (MDRD) (mL/min/1.73m^2^)	135.00 (118.25–156.75)	135.00 (112.50–157.00)	130.50 (112.00–152.00)	0.143
**Second trimester**				
Gestation weeks	25.81 ± 1.62	25.96 ± 1.56	25.88 ± 1.24	0.542
FPG (mmol/L)	4.38 ± 0.38	4.48 ± 0.48	4.47 ± 0.50	0.656
1 h PG (mmol/L)	7.31 ± 1.73	7.56 ± 2.29	7.78 ± 1.78	**0.043**
2 h PG (mmol/L)	6.17 ± 1.32	6.62 ± 1.76	6.50 ± 1.38	0.090
OGTT area	12.57 ± 2.27	13.05 ± 3.13	13.23 ± 2.43	0.067
WBC (× 10^9^/L)	9.00 ± 1.95	9.44 ± 2.18	10.07 ± 2.33	**< 0.001**
Neutrophils (× 10^9^/L)	6.50 ± 1.68	7.02 ± 1.94	7.51 ± 1.96	**< 0.001**
**Third trimester**				
Delivery time (weeks)	39.1 ± 1.26	39.0 ± 1.49	38.7 ± 1.64	0.274
HBA1C (%)	5.26 ± 0.34	5.15 ± 0.40	5.18 ± 0.35	0.719
FPG (mmol/L)	4.24 ± 0.59	4.09 ± 0.55	4.16 ± 0.90	0.396
Weight (kg)	70.46 ± 8.18	69.84 ± 8.86	70.03 ± 9.54	0.477
Weight gain (kg)	12.18 ± 4.58	12.62 ± 5.01	11.96 ± 4.57	0.876
**Pregnancy outcomes**				
GDM women (*n*, %)	18 (18.8)	32 (33.3)	46 (47.9)	**< 0.001**
Birth length (cm)	49.75 ± 0.96	49.76 ± 1.18	49.47 ± 2.09	0.708
Newborn weight (kg)	3.30 (3.03–3.52)	3.30 (3.05–3.57)	3.26 (3.01–3.55)	0.988
Macrosomia (n, %)				0.110
No	117 (97.5)	99 (91.7)	137 (95.8)	
Yes	3 (2.5)	9 (8.3)	6 (4.2)	
Fetus gender (n, %)				0.156
Male	63 (52.5)	43 (39.8)	65 (45.5)	
Female	57 (47.5)	65 (60.2)	78 (54.5)	
Comprehensive adverse fetal outcomes (n, %)				**0.016**
No	113 (94.2)	92 (85.2)	124 (86.7)	
Yes	7 (5.8)	16 (14.8)	19 (13.3)	
Cesarean section (n, %)				0.432
No	80 (66.7)	64 (59.3)	95 (66.4)	
Yes	40 (33.3)	44 (40.7)	48 (33.6)	

Data are presented as mean ± SD, median (interquartile range) or *n* (%)

**Table 3:** Comparison of parameters among three groups categorized by tertile of PR3 in the cohort study

	Lowest	Middle	Highest	*P*
PR3 (ng/mL)	Below 70.50	70.50 to 111.02	Above 111.02	/
N	196	196	197	/
**Anthropometric parameters**				
Age (years)	30.45 ± 4.17	30.68 ± 4.61	30.21 ± 4.56	0.630
Parity (n, %)				0.133
No	52 (41.3)	21 (31.3)	49 (30.4)	
Yes	74 (58.7)	46 (68.7)	112 (69.6)	
Pre-pregnancy BMI (kg/m^2^)	21.78 ± 3.19	22.19 ± 3.33	22.11 ± 3.49	0.457
**First trimester**				
Gestation weeks	11.03 ± 3.34	10.96 ± 3.07	11.02 ± 3.28	0.393
SBP (mmHg)	113.28 ± 9.82	115.46 ± 9.73	115. ± 9.91	0.436
DBP (mmHg)	70.80 ± 8.71	69.60 ± 8.16	72.82 ± 8.80	0.062
Weight (kg)	56.25 ± 8.75	56.79 ± 8.82	56.41 ± 8.44	0.273
Height (cm)	1.61 ± 0.05	1.60 ± 0.05	1.60 ± 0.06	0.892
WBC (× 10^9^/L)	8.12 ± 1.71	8.83 ± 1.98	9.10 ± 2.17	**< 0.001**
Neutrophils (× 10^9^/L)	6.02 ± 1.37	6.43 ± 1.41	6.66 ± 1.41	**0.001**
ALT (U/L)	10.00 (7.00–15.00)	10.50 (9.00–5.00)	11.00 (8.00–15.00)	0.416
BUN (mmol/L)	2.40 (2.10–2.95)	2.50 (2.08–3.03)	2.70 (2.20–3.00)	**0.029**
SCR (umol/L)	49.94 ± 8.34	51.98 ± 9.49	51.87 ± 9.10	0.097
TC (mmol/L)	4.43 ± 0.79	4.49 ± 0.77	4.58 ± 0.88	0.415
TG (mmol/L)	1.34 (1.02–1.72)	1.39 (1.09–1.85)	1.43 (1.13–1.87)	0.469
HDL (mmol/L)	1.83 ± 0.35	1.82 ± 0.36	1.82 ± 0.36	0.716
LDL (mmol/L)	2.40 ± 0.68	2.49 ± 0.63	2.54 ± 0.70	0.441
FPG (mmol/L)	4.43 ± 0.39	4.39 ± 0.49	4.39 ± 0.38	0.236
eGFR (CDK-EPI) (mL/min/1.73m^2^)	125.66 ± 9.26	123.42 ± 11.19	123.75 ± 9.99	0.169
eGFR (MDRD) (mL/min/1.73m^2^)	135.00 (118.25–157.75)	131.00 (111.00–152.25)	133.00 (112.00–153.00)	0.123
**Second trimester**				
Gestation weeks	25.93 ± 1.39	25.81 ± 1.39	25.91 ± 1.60	0.721
FPG (mmol/L)	4.42 ± 0.44	4.46 ± 0.49	4.46 ± 0.45	0.230
1 h PG (mmol/L)	7.27 ± 1.87	7.59 ± 2.04	7.83 ± 1.90	**0.002**
2 h PG (mmol/L)	6.27 ± 1.40	6.45 ± 1.62	6.57 ± 1.47	**0.004**
OGTT area	12.61 ± 2.52	13.02 ± 2.78	13.32 ± 2.57	**0.001**
WBC (× 10^9^/L)	8.70 (7.65–10.10)	9.31 (8.09–10.82)	9.73 (8.31–11.50)	**0.002**
Neutrophils (× 10^9^/L)	6.46 ± 1.67	7.16 ± 1.91	7.52 ± 2.01	**< 0.001**
**Third trimester**				
Delivery time (weeks)	38.99 ± 1.36	38.77 ± 1.55	38.75 ± 1.72	0.481
HBA1C (%)	5.27 ± 0.39	5.14 ± 0.34	5.15 ± 0.36	0.501
FPG (mmol/L)	4.16 ± 0.61	4.16 ± 0.52	4.18 ± 0.96	0.452
SBP (mmHg)	119.91 ± 10.28	121.58 ± 11.46	119.91 ± 10.30	0.266
DBP (mmHg)	74.54 ± 7.02	75.62 ± 7.78	74.77 ± 7.93	0.109
Weight (kg)	70.26 ± 8.32	70.79 ± 9.69	69.23 ± 8.61	0.200
Weight gain (kg)	12.46 ± 5.49	12.34 ± 4.16	11.87 ± 4.33	0.446
**Pregnancy outcomes**				
GDM women (*n*, %)	23 (24.0)	27 (31)	46 (47.9)	**0.011**
Birth length (cm)	49.76 ± 0.97	49.74 ± 1.13	49.44 ± 2.20	0.825
Newborn weight (kg)	3.30 (3.06–3.51)	3.30 (3.04–3.60)	3.27 (3.00–3.50)	0.651
Macrosomia (n, %)				0.200
No	100 (99.0)	50 (94.3)	116 (95.1)	
Yes	1 (1.0)	3 (5.7)	6 (4.9)	
Fetus gender (n, %)				0.873
Male	49 (48.5)	27 (50.9)	57 (46.7)	
Female	52 (51.5)	26 (49.1)	65 (53.3)	
Comprehensive adverse fetal outcomes (n, %)				0.122
No	95 (94.1)	44 (83.0)	104 (85.2)	
Yes	6 (5.9)	9 (17.0)	18 (14.8)	
Cesarean section (n, %)				0.464
No	67 (66.3)	30 (56.6)	81 (66.4)	
Yes	34 (33.7)	23 (43.4)	41 (33.6)	

Data are presented as mean ± SD, median (interquartile range) or *n* (%)

### First-trimester NE and PR3 were closely associated with neutrophil count, pre-pregnancy BMI, OGTT area, 1 h PG, 2 h PG and newborn weight

**Fig. 2:**
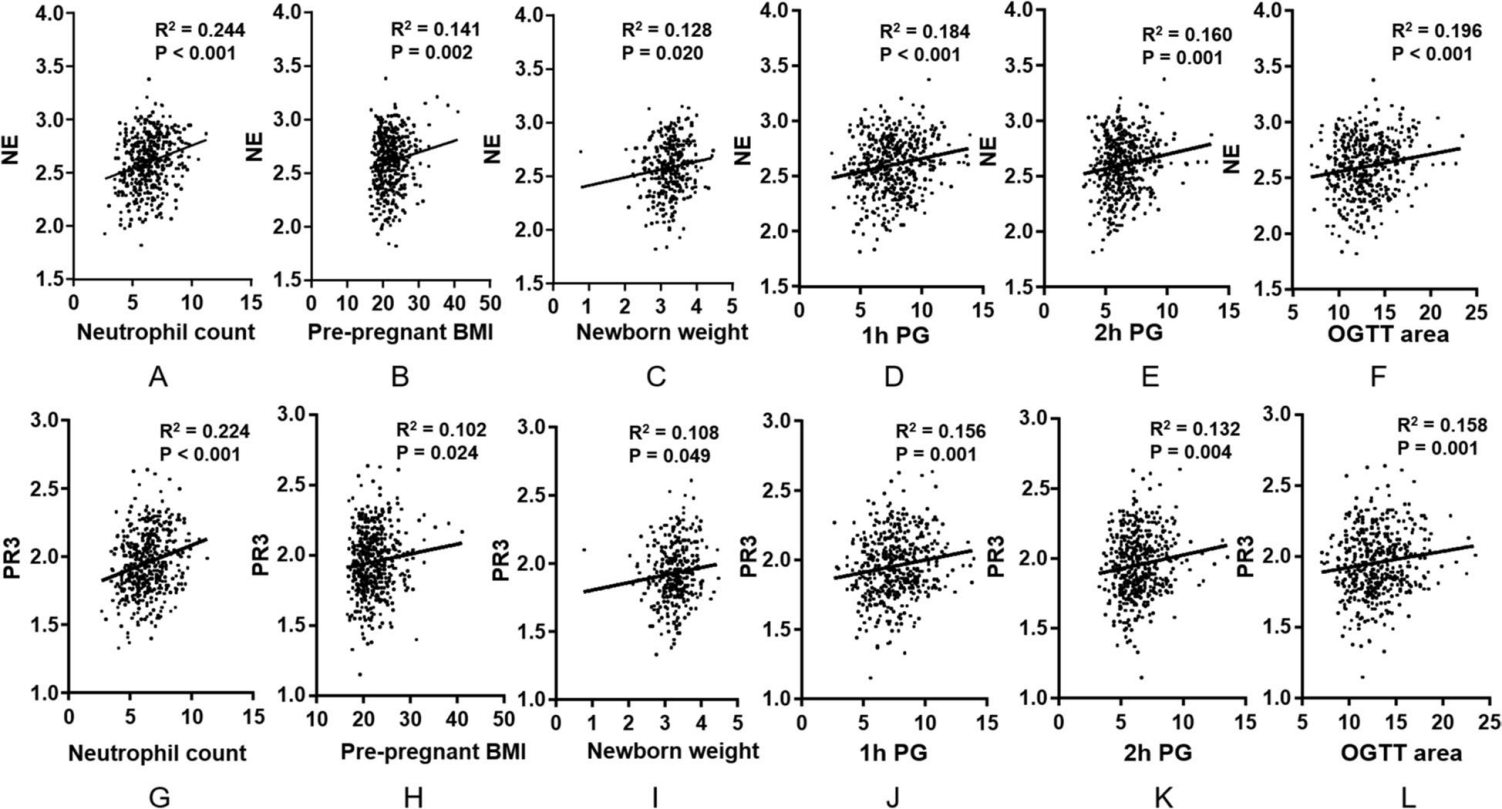
Simple linear regression analysis of association of NE and PR3 with first-trimester neutrophil count, pre-pregnancy BMI, newborn weight, 1h PG, 2h PG and OGTT area. First-trimester neutrophil count for NE β = 0.24, F (1, 453) = 28.61, adjusted *R*^2^ = 0.06; *P* < 0.001, for PR3 β = 0.22, F (1, 468) = 24.84, adjusted *R*^2^ = 0.05, *P* < 0.001 (**A** and **G**); pre-pregnancy BMI for NE β = 0.14, F (1, 472) = 9.61, adjusted *R*^2^ = 0.02, *P* = 0.002, for PR3 β = 0.10; F (1, 486) = 5.15, adjusted *R*^2^ = 0.01, *P* = 0.024 (**B** and **H**); newborn weight for NE β = 0.13, F (1, 328) = 5.49, adjusted *R*^2^ = 0.01, *P* = 0. 020, for PR3 β = 0.11, F (1, 330.00) = 3.90, adjusted *R*^2^ = 0.01, *P* = 0.049 (**C** and **I**); 1h PG for NE β = 0.18, F (1, 457) = 16.08, adjusted *R*^2^= 0.03, *P* < 0.001, for PR3 β = 0.16, F (1, 472) = 11.81, adjusted *R*^2^= 0.02, *P* = 0.001 (**D** and **J**); 2h PG for NE β = 0.16, F (1, 460) = 12.09, adjusted *R*^2^ = 0.02, *P* = 0.001, for PR3 β = 0.13, F (1, 475) = 8.38, adjusted *R*^2^ = 0.02, *P* = 0.004 (**E** and **K**); and OGTT area for NE β = 0.20, F (1, 450) = 17.97, adjusted *R*^2^ = 0.04, *P* < 0.001, for PR3 β = 0.16, F (1, 465) = 11.91, adjusted *R*^2^ = 0.02, *P* = 0.001 (**F** and **L**). NE and PR3 were log transformed for analysis

Simple linear regression analyses were performed to determine the association of NE or PR3 during the first trimester with neutrophil count, pre-pregnancy BMI, OGTT area, 1 h PG, 2h PG and newborn weight. There was a significant linear correlation of NE and PR3 with neutrophil count (Fig. 2A and G), pre-pregnancy BMI (Fig. 2B and H), newborn weight (Fig. 2C and I), 1 h PG (Fig. 2D and J), 2h PG (Fig. 2E and K) and OGTT area (Fig. 2F and L).

### Higher NE and PR3 was an independent risk factor for GDM occurrence and comprehensive adverse fetal outcomes

To determine independent risk factors for the development of GDM and comprehensive adverse fetal outcomes, tertile of NE or PR3, pre-pregnancy BMI, age, and first trimester FPG were entered into logistic regression analysis with enter selection. The risk of GDM development (OR = 2.51; *P* = 0.007) and comprehensive adverse fetal outcomes (OR = 2.98; *P* = 0.023) increased in the highest tertile NE compared with the lowest. Results were similar for PR3: the risk of GDM development increased 3.61-fold (*p* < 0.001) and comprehensive adverse outcomes 3.14-fold (*p* = 0.042), regardless of age, first-trimester FPG or pre-pregnancy BMI (Table 4).

**Table 4:** NE or PR3 were independent risk factors for development of GDM and adverse fetal outcomes in all enrolled subjects

	GDM		Comprehensive adverse fetal outcomes	
All subjects (*n* = 589)	*OR*	*P*	*OR*	*P*
NE (ng/mL)				
Lowest (< 311.49)	Reference		Reference	
Middle (311.49 to 514.96)	2.33 (1.23 to 4.44)	**0.010**	2.86 (1.16 to 7.08)	**0.023**
Highest (> 514.96)	2.51 (1.29 to 4.89)	**0.007**	2.98 (1.16 to 7.64)	**0.023**
Age (years)	1.95 (1.18 to 3.22)	**0.009**	2.02 (1.03 to 3.95)	0.042
First-trimester FPG (mmol/L)	1.27 (0.77 to 2.08)	0.350	2.04 (1.04 to 4.02)	0.038
Pre-pregnancy BMI (kg/m^2^)	3.05 (1.81 to 5.16)	**< 0.001**	1.30 (0.67 to 2.53)	0.434
PR3 (ng/mL)				
Lowest (< 70.50)	Reference		Reference	
Middle (70.50 to 111.02)	1.60 (0.87 to 2.96)	0.134	2.78 (1.05 to 7.35)	**0.039**
Highest (> 111.02)	3.61 (1.78 to 7.30)	**< 0.001**	3.14 (1.05 to 9.43)	**0.042**
Age (years)	1.65 (0.98 to 2.80)	0.061	1.88 (0.86 to 4.09)	0.112
First-trimester FPG (mmol/L)	1.30 (0.77 to 2.21)	0.333	2.05 (0.94 to 4.48)	0.073
Pre-pregnancy BMI (kg/m^2^)	3.08 (1.78 to 5.31)	**< 0.001**	1.50 (0.70 to 3.25)	0.300

### Continuous NE or PR3 in the first trimester was closely associated with the incidence of GDM and comprehensive adverse fetal outcomes

**Fig. 3:**
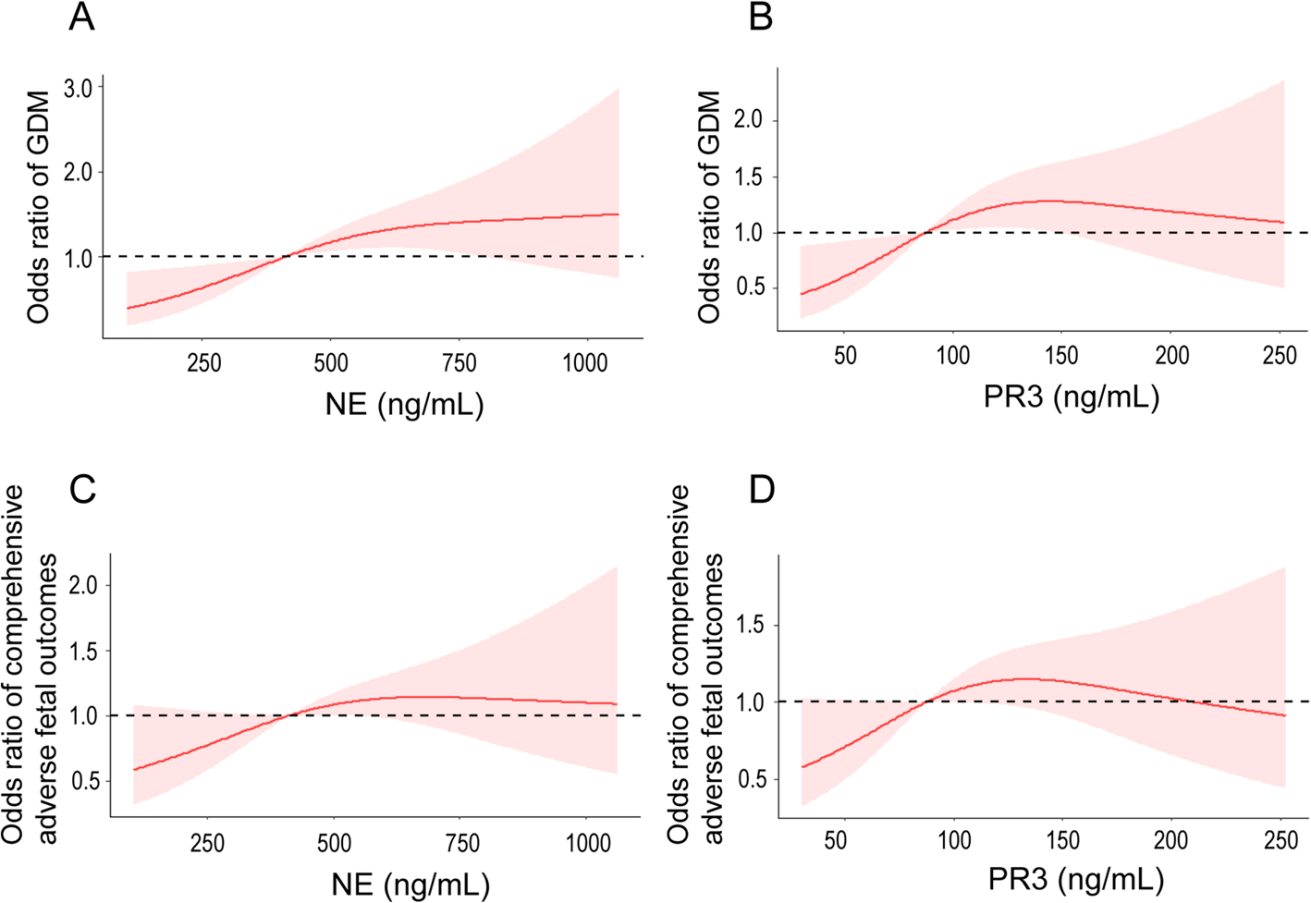
Restricted cubic spline analysis. After adjusting for age, pre-pregnancy BMI and first-trimester FPG

 After adjusting for age, first-trimester FPG and pre-pregnancy BMI, a restricted Cubic spline regression model showed a significant linear relationship between continuous NE during the first trimester and GDM occurrence and comprehensive adverse fetal outcomes, and showed a higher risk when it exceeded 417.60 ng/mL (Fig. 3A and C). A similar result was obtained for PR3 and GDM occurrence when PR3 exceeded 88.52 ng/mL, albeit showing a slight continuous decrease at the bottom of the regression line (Fig. 3B). For PR3 and comprehensive adverse outcomes showed an inverted U-shaped relationship: from 88.52 ng/mL to 208.77 ng/mL there was a higher risk of comprehensive adverse outcomes, with the highest risk at around 130.00 ng/mL (Fig. 3D).

## The expression of NE and PR3 in placental tissue was increased in women with GDM compared with those without

**Fig. 4:**
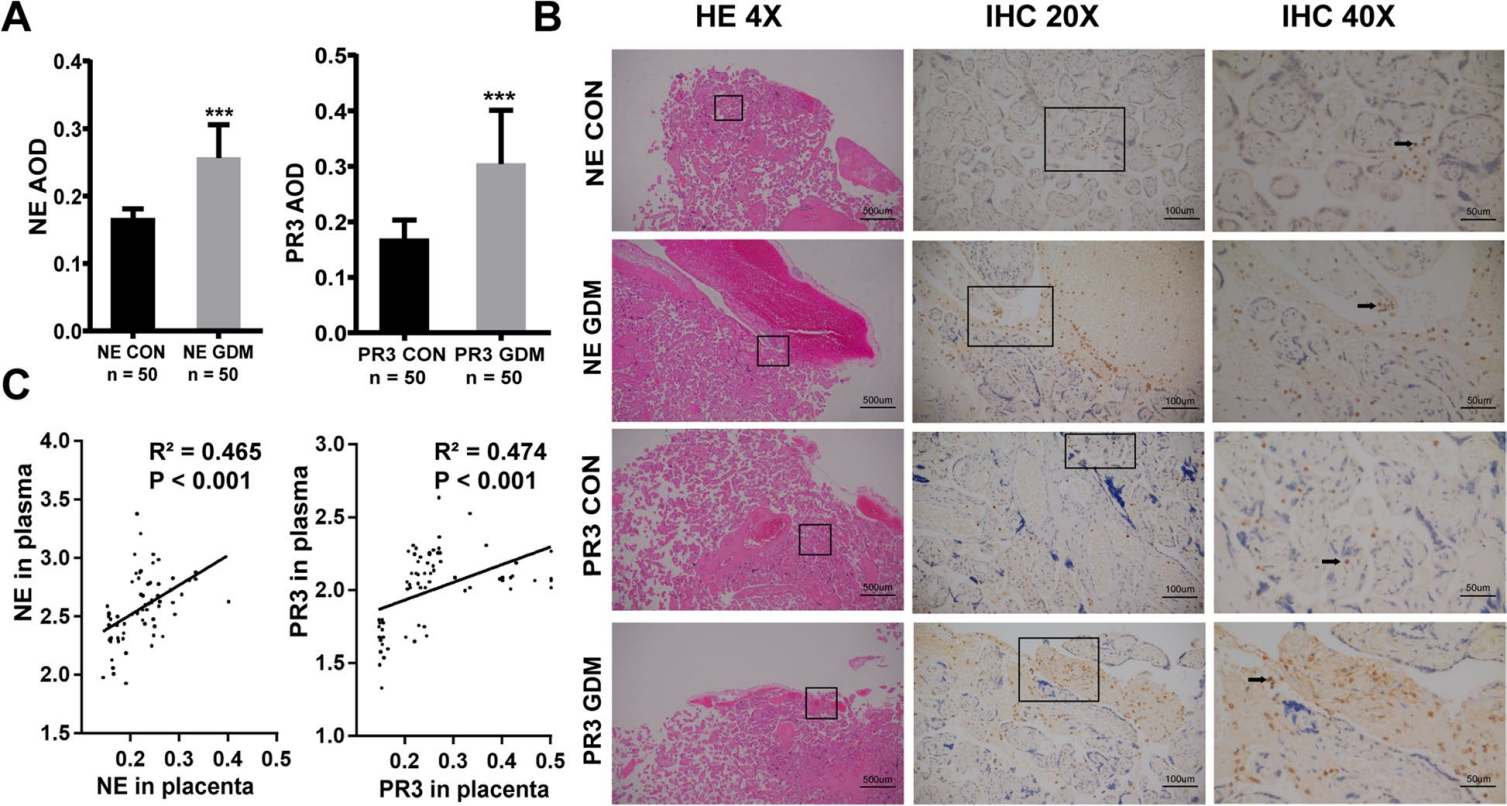
Immunohistochemistry of placental tissue in GDM cases and control women. **A** shows the difference in positive lesions of NE and PR3 in placental tissue from women with GDM and the control group through mean and standard deviation of AOD in column chart. **C** shows the correlation of circulating NE/PR3 with local NE/PR3 AOD. **B** shows NE and PR3 biomarkers in GDM cases and control group revealed by HE and IHC staining. The first picture (from left to right) shows cells in control placental tissue (4X) to locate the positive position of IHC. Next to it, the brown spot was a positive site of NE or PR3 displayed in the control group by IHC picture 20X and 40X (indicated by arrows). GDM group is shown under the relevant control group. ***, *P*<0.001

 As shown in Fig. 4, NE positive area and PR3 positive area was significantly higher in GDM placental tissue than non-GDM tissue shown by AOD (NE 0.26 ± 0.05 vs. 0.17 ± 0.01 ng/mL, *p* < 0.001; PR3 0.30 ± 0.09 vs. 0.17 ± 0.03 ng/mL, *p* < 0.001, respectively). Typical histological images of both H&E staining and immunohistochemical staining with NE and PR3 are presented in Fig. 4. Furthermore, correlation of circulating NE/PR3 with local NE/PR3 AOD was analyzed and revealed that circulating levels of NE/PR3 were closely associated with local NE/PR3 expression (Fig. 4).

## Discussion

Several studies have identified homeostatic pro-inflammatory and anti-inflammatory mechanisms in pregnant women. GDM is a chronic inflammatory process, and insulin resistance and overweight can cause an imbalance in the regulation of inflammation [14–16].

Many studies have reported that white blood cell and neutrophil counts are often elevated during pregnancy and related inflammatory markers are significantly increased compared with the nonpregnant state [17, 18]. A growing number of studies have described the central role of inflammation in GDM development [5, 15, 17, 18]. In our previous cohort study, women who developed GDM had a much higher neutrophil count in the first trimester than those who did not [5]. NSPs are stored in the azurophilic granules of neutrophils. Upon neutrophil activation, they are released from the granules and can activate cytokines in the neutrophil cytosol or in the extracellular space, then result in metabolic disturbance [19]. We hypothesized that neutrophil count plays a key role in this programming process via NE and PR3. Moreover, in spline regression analysis, we found an inverted U-shaped linear correlation between comprehensive adverse fetal outcomes and PR3. This may explain the lack of a statistically significant difference between PR3 tertiles.

Recently, only few studies have focused on the effect of NSPs in metabolic disease. Both NE and PR3 mediate a chronic inflammatory state by activating the pro-form of tumor necrosis factor (pro-TNF) and the pro-form of interleukin-1β (pro IL − 1β) [20, 21]; activating specific cellular receptors (NE and PR3 have been proposed to activate proteinase-activated receptor 2 (PAR2) and phospholipase C (PLC) leading to translocation of nuclear factor-κB (NF-κB)) [22]. In HFD obese mice, the activity of NE increased, revealing the involvement of NE in the development of obesity-related metabolic complications [12]. In addition, some researchers have reported that neutrophil activity was stimulated with consequent release of NE in GDM women in the second trimester in vitro [23, 24].

In our prospective cohort study, NE and PR3 were increased significantly in women with GDM in the first trimester, and their strong association with GDM development persisted after matching for pre-pregnancy BMI and age. The occurrence of GDM and comprehensive adverse fetal outcomes gradually increased across tertitles of NE and PR3. To explore risk factors for GDM development and adverse fetal outcomes, we included NE and PR3 after adjusting for age, pre-pregnancy BMI, and FPG in the first trimester in binary regression analyses. As expected, an increase in NE and PR3 was an independent risk factor for GDM occurrence and comprehensive adverse fetal outcomes.

In the first trimester, although NE and PR3 level played an important role in GDM occurrence and progression of adverse pregnancy outcomes, it did not have a significant impact in the second trimester compared with non-GDM women. This may suggest that a stronger inflammatory activation state in early pregnancy results in abnormal glucose regulation later on. It also suggests that the overactive inflammatory state in early pregnancy reduces as pregnancy advances. Nonetheless potential pathological and physiological changes may have occurred during early pregnancy despite a normal blood glucose level so it is important to identify potential risks during early pregnancy and to reduce the risk of later GDM development and pregnancy adverse outcomes.

Both PR3 and NE concentration were positively correlated with neutrophil count during early pregnancy and pre-pregnancy BMI, confirming that both PR3 and NE are released during the inflammatory state and might contribute to it. As expected, neutrophil count increased during the first trimester in GDM women, consistent with results of our previous retrospective cohort study [5]. A study with a murine diabetes model revealed that exogenous NE led to insulin resistance in hepatocytes and contributed to inflammation-induced metabolic disease [25]. NE has been shown to lead to an exacerbation of insulin resistance via the degradation of insulin receptor substrate 1(IRS1) and a decrease in the abundance of the glucose transporter glucose transporter type 4 gene protein, resulting in diminished glucose uptake [24, 26].

Finally, in placental tissue, semi-quantitative immunohistochemical staining analysis indicated that the expression of NE and PR3 was increased. Of note, these placental alterations are frequently evident and closely associated with the development of GDM and pregnancy outcomes [24]. The study revealed that circulating levels of NE/PR3 were closely associated with local NE/PR3 expression, suggesting that infiltration of NE in placental tissue can be attributed to an increase in its circulating level. Nonetheless the connection should be validated with a larger sample size and reliable animal models.

There were some limitations to this study. First, all subjects were derived from one center and this may have led to biased results. Second, because most recruited women with GDM had mild symptoms, the occurrence of macrosomia did not present an obvious association with NE or PR3. Finally, our results showed that NE (R2 = 0.244, *P* < 0.001) and PR3 (R2 = 0.224, *P* < 0.001) were significantly associated with neutrophil (NEU) count. Nonetheless although NE/PR3 are mainly released when neutrophils are specifically stimulated, they are also derived from other cells or tissues such as granulocytes, monocytes, and mast cells [19, 27], or granulocytic myeloid derived suppressor cells (MDSCs) [28]. So NE/PR3 did not equate to neutrophils. We found that NE and PR3 were closely associated with GDM development and adverse fetal effects but it is acknowledged that a mechanistic insight into the potentially pathophysiological involvement of the increased NE/PR3 is lacking in this clinical study.

## Conclusions

This study showed that first-trimester NE and PR3 were closely associated with GDM development and comprehensive adverse fetal outcomes. Spline regression showed a significant increased risk of GDM occurrence and comprehensive adverse fetal outcomes when serum NE concentration exceeded 417.60 ng/mL. A similar result was obtained for PR3 and GDM occurrence when PR3 exceeded 88.52 ng/mL. Enriched NE and PR3 content in GDM placental tissue may explain in part the potential mechanisms that underlie the development of GDM.

### Supplementary Information

**Additional file 1:** **Supplemental Table 1.** The distribution of Mode of delivery in comprehensive adverse fetal outcomes and without.

## Data Availability

The datasets generated during and/or analyzed during the current study are not publicly available but are available from the corresponding author on reasonable request.

## Abbreviations

NSPs: Neutrophil serine proteases
GDM: Gestational diabetes mellitus
HbA1c: Glycosylated hemoglobin
BMI: Body mass index
SD: Standard deviation
TC: Total cholesterol
TG: Triglyceride
HDL: High density lipoprotein
LDL: Llow density lipoprotein
ALT: Alanine aminotransferase
BUN: Blood urea nitrogen
SCR: Serum creatinine
eGFR: Estimated glomerular filtration rate
FPG: Fasting plasma glucose
WBC: White blood cell
NE: Neutrophil elastase
PR3: Proteinase-3
SBP: Systolic blood pressure
DBP: Diastolic blood pressure
OGTT: Oral glucose tolerance test
1h PG: 1 hour plasma glucose
2h PG: 2 hour plasma glucose
