# Supplementary material for: Elevated first-trimester neutrophil elastase and proteinase 3 increase the risk of gestational diabetes mellitus and adverse fetal outcomes
Source: Reprod Biol Endocrinol. 2024 Jan 2;22:2. doi: 10.1186/s12958-023-01170-x (PMC10759696; doi:10.1186/s12958-023-01170-x)
Supplement: Supplementary file 1 — Additional file 1: Supplemental Table 1. The distribution of Mode of delivery in comprehensive adverse fetal outcomes and without. [file 12958_2023_1170_MOESM1_ESM.docx]

**Supplemental Table 1.** T**he distribution of Mode of delivery in comprehensive adverse fetal outcomes and without**

| **Mode of delivery (n, %)** | **Without comprehensive adverse fetal outcomes** | **Comprehensive adverse fetal outcomes** | ***P*** |
| --- | --- | --- | --- |
| Cesarean section | 115 (33.5) | 24 (51.1) | 0.016 |
| Eutocia | 228 (66.5) | 23 (48.9) |  |
